# Feedback on Mental State Inferences Improves Accuracy and Awareness

**DOI:** 10.1177/17470218251404419

**Published:** 2025-11-24

**Authors:** Bryony Payne, Geoffrey Bird, Caroline Catmur

**Affiliations:** 1 Department of Psychology, Institute of Psychiatry, Psychology and Neuroscience, King’s College London, UK; 2 Department of Experimental Psychology, University of Oxford, UK; 3 Centre for Research in Autism and Education, Institute of Education, University College London, UK

**Keywords:** mentalising, theory of mind, mental state inference, out-group, feedback

## Abstract

Accurate inference of the mental states of others is essential for successful social interaction. Concerningly, previous work shows that humans are less accurate when inferring the views of out-group members relative to in-group members, but are unaware of this difference in accuracy. Across two studies (Experiment 1: *n* = 142; Experiment 2: *n* = 90), we asked whether feedback on the accuracy of mental state inferences could increase the accuracy of, and/or recalibrate participants’ confidence in the accuracy of, mental state inferences for outgroup members. Feedback specific to individual targets significantly improved the accuracy of inferences when inferring those targets’ views for both in-group and out-group members but did not generalise to other group members. Furthermore, participants were able to use feedback to calibrate their confidence in the accuracy of their out-group inferences. These results demonstrate that, with targeted feedback, people are more able to understand the minds of both in-group and out-group members and become more aware of their ability to do so.

## Introduction

To have a harmonious society, people need to understand each other. Yet people are more divided on key social issues than ever before (McCoy & Somer, 2019; McCoy et al., 2018). Polarisation is increasing in most Western societies (Boxell et al. 2020; Iyengar et al., 2019) and it may be underpinned, in part, by people’s reduced ability to understand people who are different from them (Payne et al., 2024). Differences may include factors such as religion, political views, or socio-economic status, such that different individuals are classified as part of an ‘out-group’, as opposed to similar individuals who share characteristics and who are classified as part of an ‘in-group’. This classification is thought to arise from each person’s self-identity: self-categorisation theory (Turner & Oakes, 1986) proposes that individuals categorise themselves at different levels, from the personal to the social, and that these categorisations give rise to different identities as a function of the context. These categorisations, in turn, influence people’s attitudes to out-group members: people demonstrate in-group favouritism and out-group discrimination, even when the group identity is formed on the basis of ‘minimal’ or arbitrary criteria (Tajfel et al., 1971). These attitudes may then impact the ability to understand out-group members by increasing the tendency to consider the out-group as a homogeneous whole (Linville & Jones, 1980).

To understand each other, people must make accurate inferences about others’ mental states, including their beliefs, desires, and intentions. This fundamental cognitive ability is known as the theory of mind or mentalising (Wellman, 2004). Yet previous work has shown mixed effects regarding differences in mental state inference for in- and out-group members. For example, Gönültaş et al. (2019) found that children were less accurate and/or used fewer mental state words when reasoning about out-group members compared to in-group members in a ‘strange stories’ mentalising task. In contrast, undergraduate participants indicated that an out-group protagonist was more likely to act in accordance with a false belief than an in-group protagonist (Todd et al., 2011). This finding was interpreted as demonstrating ‘better’ mentalising for the out-group protagonist than the in-group protagonist, though this effect could instead be due to increased egocentricity for in-group members (Samuel, 2023). Moreover, this false-belief task does not have a ‘correct’ answer: the mental state of the protagonist is not known, and participants incorporate information about the protagonist’s traits and other beliefs (e.g. the protagonist’s knowledge of the other character’s deceptiveness) into their judgements of the protagonist’s likely actions (Conway et al., 2020). Therefore, increased ratings for acting in accordance with a false belief for an out-group protagonist could in fact reflect *less* consideration of their mental state. More recently, Payne et al. (2024) demonstrated that people are no less *willing* to consider the minds of out-groups when needed, but are significantly less accurate when doing so. In spite of this reduced accuracy, people remained highly confident that they were able to predict the views of an out-group member. Thus, there is a misalignment between ability and confidence, which reflects the relatively poor awareness people have of their (in)ability to understand out-group members.

The reduced ability to understand out-group members may manifest both in terms of poor accuracy when predicting the views of individual group members and in an underestimation of the variance of the range of views held across the group. It has been suggested (Conway et al., 2019) that both effects may arise from reduced experience with out-groups. People are more likely to interact with similar others (i.e. in-groups of the same ethnicity, gender, social class, or occupation; McPherson et al., 2001, 2006), and an increased number of interactions is thought to lead to a more accurate representation of the variety of views within a group (Konovalova & Le Mens, 2018, 2020; Linville et al., 1989). Further, with limited direct experience, one’s representation of out-groups may be relatively more influenced by indirect means, such as through information in the media, which frequently portrays out-groups negatively and uniformly (as a ‘threatening homogeneous collective’; Innes, 2010, p. 461).

The consequence of having inaccurate representations of out-groups is that they give rise to incorrect assumptions about out-groups and their mental states. People typically overestimate the extremity of the views of out-groups (Ahler & Sood, 2018; Alesina et al., 2018; Levendusky & Malhotra, 2016). For instance, those who support the Republican party overestimated the extremity of Democrat views on taxes, abortion, gay rights, and racial policy (Ahler & Sood, 2018). These incorrect assumptions about the extremity and homogeneity of out-group views may fuel affective polarisation, whereby distrust, dislike, and hostility towards the out-group increases (Abramowitz & McCoy, 2019; Bliuc et al., 2021; Druckman & Levendusky, 2019), and people are less likely to either help out-group members (Kunstman & Plant, 2008; D. A. Saucier et al., 2005) or to value their lives as highly (Pratto & Glasford, 2008). In addition, increased polarisation results in increased distancing from the outgroup, producing less experience with the outgroup, and increasingly inaccurate representations of out-group views as described above. People become less willing to engage with dissimilar others, preferring not to work with, live near to, or even sit near to an out-group member whose views differ from their own (Skitka et al., 2005, 2013), meaning that there is less opportunity to have one’s assumptions about the beliefs of the out-group tested.

One of the key interventions that has been used to improve intergroup attitudes is perspective-taking, defined as actively considering the other’s subjective experiences and their mental states (see Todd & Galinsky, 2014, for review). Perspective-taking may exert its positive effects on intergroup attitudes in a range of ways, including strengthening links between the self and the out-group, with resulting positive self-evaluations being transferred to the out-group (Todd & Burgmer, 2013); and by helping one to individualise particular out-group members (Leyens et al., 2003). However, perspective-taking may not actually improve the accuracy of mental state inference (see Eyal et al., 2018, for a comprehensive test of this hypothesis), in particular where the other person’s mental states are dissimilar to one’s own. It therefore seems critical to investigate alternative ways to correct inaccurate representations of out-group mental states.

Despite the importance of obtaining an accurate representation of out-group mental states, it remains unknown whether, even with the opportunity to receive feedback, people would be willing to update their beliefs about out-groups. Previous studies have suggested that feedback-based interventions can improve people’s performance at person perception (see Blanch-Hartigan et al., 2012, for review), with training and/or instruction facilitating abilities such as deception detection and empathic accuracy, which depend on processes such as perceiving non-verbal cues from facial expressions and biological motion, and emotion recognition. However, such training does not focus specifically on improving mental state inference. Examining mental state inference more closely, previous studies have suggested that feedback-based interventions can improve children’s performance on false belief tasks (Guajardo et al., 2013) and perspective taking in adults (Damen et al., 2021). Further, Marangoni et al. (1995) demonstrated that inferences about a target’s mental and emotional states could be improved with corrective feedback, but found the effect was smaller than that afforded by exposure to – and experience with – the target over time. Participants also showed very poor awareness of their own (in)ability to accurately understand the target’s mental and emotional states, such that their perception of their accuracy was uncorrelated with their actual accuracy.

Critically, however, these prior feedback-based studies all assume that these abilities are stable and constant across targets as opposed to target-dependent. That is, none allow for the possibility that inference accuracy may differ for in-group and out-group minds (Conway et al., 2020), and, moreover, none allow for the fact that people may be differentially aware of their own (in)ability to make accurate inferences depending on who the target is (Payne et al., 2024). Indeed, if accuracy – and awareness of one’s accuracy – differ depending on the target, the effect of feedback on accuracy and on awareness may also differ depending on the target. For instance, learning may be selective: people have a bias for learning facts that corroborate their world views relative to those that challenge them (Jerit & Barabas, 2012). It is essential, therefore, to test the efficacy of feedback on mental state inference about both in-group and out-group members. One starting point for improving the accuracy of mental state inference for out-groups, specifically, could be to address the misplaced confidence that people have in their (inaccurate) inferences (Payne et al., 2024). Becoming aware that one is not, in fact, representing an out-group member’s mental states correctly might, in due course, lead people to seek more information about the individual’s actual beliefs, eventually resulting in increased accuracy.

Accordingly, we presented participants with real-world individuals (‘targets’) whose attitudes on various social and political statements rendered them either in-group (i.e. they agreed with the participant) or out-group (i.e. they disagreed with the participant; We note that there are many bases upon which individuals may develop a group identity, including demonstrations of ‘minimal groups’ formed on the basis of ‘fairly irrelevant classification’ (Tajfel et al., 1971); here, following Payne et al. (2024), we use politically polarising statements to determine group classification). Across two studies, we examined two types of feedback, assessing whether either was effective at: (a) improving participants’ accuracy in understanding others; (b) changing participants’ confidence in their mental state inferences or (c) improving the degree to which participants’ confidence tracked their accuracy when inferring in-group and out-group mental states. In Experiment 1, we investigated whether feedback about the views of individual targets would generalise to more accurate inferences about other targets in the group; whereas in Experiment 2, we tested whether target-specific feedback would improve inferences about that specific target.

## Experiment 1

In Experiment 1, we provided one group of participants with feedback on a subset of their incorrect mental state inferences about individual in- or out-group targets. The feedback highlighted that their initial inference was incorrect and provided the target’s true belief. We assessed whether this type of feedback – learning about a subset of individual minds from both the in- and out-groups – would generalise to an improvement in one’s mental state inferences about additional, novel, individuals from those groups.

We hypothesised (see pre-registration linked below) that overall, accuracy, confidence, and awareness (accuracy-confidence correlation) would be higher for in- than out-group targets. Regarding the effect of feedback, if feedback generalises to novel individuals, we hypothesised that accuracy should be higher for both in- and out-group targets within the feedback condition compared to the no-feedback condition. We also hypothesised that the difference in inference accuracy between in- and out-group targets might be less for participants in the feedback condition compared to the no-feedback condition. We hypothesised that confidence would differ and that awareness would be higher for participants in the feedback compared to the no-feedback condition, and that these effects might also manifest as an interaction between feedback condition and target group status.

### Methods

#### Transparency and Openness

We report how we determined our sample size, all data exclusions (if any), all manipulations, and all measures in the study, and the study follows the Journal Article Reporting Standards. Data and analysis scripts for Experiment 1 and 2, along with the pre-registrations containing design and analysis plan for each experiment, are available at: https://osf.io/d8xuw/.

#### Participants

In Experiment 1, following our pre-registered sampling strategy, we initially recruited 173 participants to Part 1, aiming to obtain a sample of 150 participants eligible for Part 2 after exclusion criteria had been applied. This sample size of 150 was determined by a power analysis in G*Power for a 2-way mixed analysis of variance to assess an interaction between Group Status (in-group vs. out-group) and Feedback (feedback vs. no feedback). Our goal was to obtain .95 power to detect a small-to-medium effect size of .3 at the standard .05 alpha probability. This sample size of 150 was achieved, however, after the exclusion criteria were applied to Part 2, a final sample size of 142 participants remained (mean age = 45.91 years, *SD* = 12.82 years, age range = 23–79, 71 female and 71 male) and it was not possible to replace excluded participants given the between-group balancing that was required. According to the G*Power analysis, only 90 participants were needed if powered to 0.80 (rather than to 0.95), so 142 participants were deemed sufficient.

All participants were recruited online via Prolific (www.prolific.ac), self-reported as neurotypical, with English as their primary language, and the United States as both their country of birth and current place of residence. Participants reported no significant visual impairments, mild cognitive impairments, or dementia. Finally, participants had to have joined Prolific before 2020 and have a current approval rate of over 90% to ensure data quality. All participants were tested online using Gorilla (gorilla.sc, Anwyl-Irvine et al., 2019). None had taken part in any pilot studies associated with this project and, upon completion of the experiment, were paid for their participation. Ethical approval was obtained from the local Health Faculties Research Ethics Subcommittee, all research was performed in accordance with the regulations of the Declaration of Helsinki and, as such, informed consent was obtained from all participants prior to testing.

#### Stimuli Development

A novel stimulus set was created based on the Survey of Beliefs and Opinions (G. Saucier, 2018), and responses to this survey constituted the ground-truth against which accuracy was assessed. The original survey included statements such as ‘We must ensure that women have access to legal abortions’, ‘I feel that people get what they deserve’ and ‘Only adults who know how to read and write should be allowed to vote’. Thus, a person’s previous responses to these statements constitute their mental states, that is, their propositional attitudes (Butterfill & Apperly, 2013; Leslie, 1987). In the current experiment, a set of 56 pairs of statements was selected based on the correlation between previous responses across these statements in a sample of 703 American respondents (G. Saucier, 2018). Specifically, we selected 56 pairs of statements (see Supplemental Materials for full list of statements), where each pair contained an initial ‘starting’ statement and a correlated ‘target’ statement (*r*_M_ = .34, *r*_SD_ = .09). For each pair, we selected a ‘target-mind’ – a previous respondent whose responses across these statements were set as the ground-truth against which new participants in the current experiment would be assessed in terms of accuracy at inferring their responses. To ensure this ground truth was not idiosyncratic, we selected target minds whose responses across the statements in their pair were the modal response for all the previous respondents.

### Procedure: Part 1

#### Part 1, Task 1: Assessing Group Status

The first task was completed to determine participants’ group status in relation to each of the target-minds they would be asked to make mental state inferences about. Participants were presented with 56 of the starting statements in randomised order and were asked to state their agreement/disagreement with the statement on a 5-point Likert scale. Across all the trials and tasks, the scale was as follows: 1 = *strongly disagree*; 2 = *slightly disagree*; 3 = *neither agree nor disagree*; 4 = *slightly agree* and; 5 = *strongly agree*. This scale was chosen to align with responses in the original sample (G. Saucier, 2018) from which these statements were selected. The participant’s response was compared to the target-mind’s response, and the difference in agreement between the two was used to index group status (i.e. whether the target-mind was in-group or out-group with respect to the current participant). When the participant and target-mind had a difference in agreement score of 0 or 1 (i.e. the participant gave the same response to the target-mind or matched the target-mind on either agreeing or disagreeing with the statement, but with different strengths of conviction, e.g. slightly agree vs. strongly agree), they were defined as in-group. Trials in which participants gave a neutral response (i.e. that they neither agreed nor disagreed) were not coded (see ‘Exclusion criteria’). Finally, when the participant and target-mind had a difference in agreement score of either 2, 3, or 4, they were defined as out-group.

On each trial, participants were not explicitly made aware of this in- or out-grouping, thus any effect of group status is a consequence of the participant’s own perception of differences between themselves and the target-mind.

#### Part 1, Task 2: Predicting the Views of Others

This task measured participants’ accuracy in inferring the mental states of others, their confidence on each trial, and how aware participants were of their ability to make accurate inferences. This latter measure was operationalised as the correlation between actual inference accuracy and confidence in the accuracy of that inference. On each of the 56 trials, presented in randomised order, participants were presented with a target-mind’s response to an initial ‘starting’ statement. For instance: ‘Participant 437 [the target-mind] said that they *strongly disagree* that they believe in the superiority of their own gender’. Based on this information about one of their mental states, participants were asked to predict the target-mind’s response to a second target statement on a scale of 1–5, whereby 1 denoted ‘*strongly disagreed*’ and 5 denoted ‘*strongly agreed*’. For instance, participants were asked whether they thought Participant 437 thought that ‘We should ensure that no one is denied a job due to prejudice’. No feedback was given. Participants were then asked how confident they were in their answer and could respond on a scale of 0–100, where 0 = *Not confident at all* and 100 = *Extremely confident*. Participants were told that there was the possibility of a bonus payment if they achieved over 75% accuracy in this task.

Finally, five ‘attention check’ trials were included in each task. In these trials, participants were not presented with a statement to give their opinion or prediction on, but instead were told what response to select, for example, ‘This is an attention check. Please click on the “strongly agree” response option’. Completion of these tasks ended the first part of the experiment.

#### Exclusion Criteria for Part 1

For Part 1 of the experiment, the pre-registered criteria stated that: (i) whole datasets for participants who indicate that they themselves ‘neither agree nor disagree’ on >25% of the starting statement trials would be excluded and their data replaced. This exclusion criterion accounted for 2 exclusions; (ii) trials on which participants responded in <1,000 ms and >30,000 ms would be excluded, and, relatedly, (iii) whole datasets would be removed for participants who had more than 25% of trials fall under this exclusion criteria. This exclusion criterion accounted for 1 participant-level exclusion and <1% of trials. (iv) Whole datasets for participants who failed to achieve at least 80% correct in attention check trials would be excluded and their data replaced, as this suggests a lack of engagement with the task. This exclusion criterion accounted for 4 exclusions.

Finally, it was essential that participants had sufficient trials to continue to Part 2. Specifically, at least 8 inaccurate in-group trials, 8 inaccurate out-group trials, 4 accurate in-group trials, 4 accurate out-group trials were needed, to then be divided between the feedback trials and subsequent test trials. This was the minimum number of trials for the required analysis of test trials. This exclusion criterion accounted for 16 exclusions. Note that originally, before collecting the Part 1 data, we pre-registered that participants would be excluded if they did not have 8 trials in each of the above categories; however, this would have meant excluding and retesting 133 participants, which was not financially viable. Therefore, before collecting the Part 2 data, we updated our pre-registration to accommodate reduced numbers of trials, as specified here. Full details can be found on the pre-registration linked above.

In order to balance participants across the between-subjects condition (Feedback vs. No feedback), subsets of trials per participant were picked, which would allow for matching both across the feedback conditions, but also by the within-subjects factor of Group Status. Therefore, at the group level (Feedback vs. No feedback) participants were matched by their performance in Part 1 on: in-group and out-group accuracy, in-group and out-group distance to answer, in-group and out-group confidence, and levels of awareness. All 150 eligible participants were invited back to complete Part 2, and all agreed.

### Procedure: Part 2

#### Part 2, Task 1: Feedback Manipulation

Participants were randomly assigned to either the Feedback or No Feedback condition and were told they would be reminded about a randomised subset of their earlier trials. Specifically, in the ‘No feedback’ condition, participants were reminded of their answers on 4 inaccurate in-group trials and 4 inaccurate out-group trials but were not told about whether they had been accurate or not. In the ‘Feedback’ condition, participants were reminded of their answers on 4 inaccurate in-group trials and 4 inaccurate out-group trials but were additionally told these answers had been inaccurate, and what the correct answer should have been. This allowed participants to both know that they had been incorrect, and understand the magnitude of their error. Additionally, it allowed participants to update their representation of the individual target to include the target’s genuine belief. All trials were presented in randomised order.

#### Part 2, Task 2: Testing the Effect of the Feedback Manipulation

All participants underwent 16 test trials. On each, they were reminded of their previous answer to that trial in Part 1. Although participants were not told whether they had previously been correct on these test trials or not, the 16 trials they were reminded of comprised their answers to 4 previously accurate in-group trials, 4 previously accurate out-group trials, 4 previously inaccurate in-group trials and 4 previously inaccurate out-group trials. Trials were presented in a randomised order. On each trial, participants were told they could change their answer if they wished and were presented with a screen asking them whether they wanted to ‘Keep’ or ‘Change’ their answer. If participants selected to change their answer, the updated answer was recorded on the same scale as in Part 1: on a scale of 1–5, whereby 1 denoted ‘*strongly disagreed*’ and 5 denoted ‘*strongly agreed*’. Regardless of whether participants opted to ‘Keep’ or ‘Change’ their answer, they were asked to provide an updated confidence score on their final answer, again on a scale of 0–100, where 0 = *Not confident at all* and 100 = *Extremely confident*. To note, each participant was presented with an individualised subset of their previous trials. This was necessary to match participants’ Part 1 performance across the two conditions. As in Part 1, participants were told that there was the possibility of a bonus payment if they achieved over 75% accuracy in this task. Finally, ‘attention check’ trials were included here too, following the same structure as outlined above in Part 1.

#### Exclusion Criteria for Part 2

For Part 2 of the experiment, the exclusion criteria were as follows: (i) whole datasets for participants who failed to achieve at least 80% correct in attention check trials would be excluded. This accounted for no exclusions; (ii) whole datasets would be removed for participants who, on more than 25% of the trials in which they chose to ‘change’ their answer, actually selected the same response as before, as this would suggest a lack of engagement with the task. This accounted for no participant-level exclusions but, further to the pre-registered criteria, 1.4% trials were removed on this basis; (iii) trials on which participants responded in <1,000 ms and >30,000 ms would be excluded and, relatedly, whole datasets would be removed for participants who have more than 25% of trials fall under this exclusion criteria. This accounted for 1.2% of trials and 8 participant-level exclusions. After these exclusion criteria had been applied, the final sample size was 142 participants.

#### Analysis Strategy

Trials from Part 1 of the experiment were used to divide participants into matched groups (either the Feedback or No Feedback condition), based on their baseline performance. Baseline performance was analysed to determine whether previous findings by Payne et al. (2024) about the accuracy of out-group mental state inferences were replicated.

At both baseline and test, three variables were measured. First, the accuracy of participants’ mental state inferences. Accuracy was calculated as the percentage of correct responses, and only cases where participants selected the target-mind’s exact response were coded as correct, while all other responses were coded as incorrect. Second, participants’ confidence in their final answer and, third, participants’ awareness of their own ability to make an accurate inference. Awareness was operationalised as the correlation between each participant’s accuracy and their confidence scores. Calculation and analysis of dependent variables was as follows:

Accuracy: Baseline accuracy was measured via the 56 trials each participant underwent in Part 1. A generalised linear mixed model was used to assess whether participant accuracy differs as a function of the within-subjects factor of Group Status (in-group vs. out-group). The models included ‘participant’ as a random intercept.

To analyse the effect of feedback, we assessed accuracy on the 16 test trials in Part 2 as a function of the between-subjects factor of Feedback (Feedback vs. No Feedback) and the within-subjects factor of Group Status (in-group vs. out-group). Specifically, we ran generalised linear mixed-effect models to assess each of the main effects and their interaction on participants’ accuracy scores. The models again included ‘participant’ as a random intercept.

Confidence: Baseline confidence was assessed via a linear mixed model to determine whether participants’ confidence differed as a function of the within-subjects factor of Group Status (in-group vs. out-group). The models included ‘participant’ as a random intercept.

To test the effect of feedback on confidence, further linear mixed models were run on test trials only, examining the main effects of Group Status and Feedback and their interaction on test trials. The model included ‘participant’ as a random intercept.

Awareness: Baseline awareness scores were calculated as the correlation between each participant’s accuracy scores and confidence scores for both in-group and out-group trials in Part 1. These correlations were *z*-transformed and entered into a paired samples *t*-test to test for any difference between the correlations as a function of Group Status.

The effect of feedback on awareness was calculated using the correlation between each participant’s accuracy scores and confidence scores for both in-group and out-group trials on the test trials. After *z*-transforming these scores, they were entered into a mixed ANOVA to determine any difference between the correlations as a function of Group Status or Feedback. These data were analysed with *t*-tests/ANOVA rather than linear mixed models because the data structure comprises one *z* value per cell of the design, per participant, making it unsuitable for mixed-model analysis.

### Results

#### Baseline Performance (Part 1)

Participants’ inference accuracy, confidence and awareness scores at baseline (i.e. before the feedback manipulation) are reported in Table 1.

**Table 1.** Mean (*SD*) Percentage of Participants’ Accuracy, Confidence and Awareness at Baseline in Experiment 1.

	Measure		
Group Status	Accuracy (%)	Confidence	Accuracy- Confidence Awareness (*z*-scores)
In-group	44.45 (13.3)	72.99 (11.2)	0.32 (0.23)
Out-group	38.3 (13.0)	70.85 (12.7)	0.16 (0.27)

#### Baseline Accuracy

The generalised linear mixed model revealed a significant main effect of Group Status (In-group vs. Out-group) on participants’ accuracy (*X*^2^(1) = 30.158, *p* < .0001), such that accuracy was significantly greater for in-group members relative to out-group members.

#### Baseline Confidence

The linear mixed model revealed a significant main effect of Group Status (In-group vs. Out-group) on participants’ confidence (*X*^2^(1) = 27.408, *p* < .0001), such that people were more confident in their own ability to predict the views of in-group members than out-group members.

#### Baseline Awareness

A paired samples *t*-test comparing the *r*-to-*z* transformed accuracy-confidence correlations for in-group and out-group trials was significant (*t*(138) = 5.255, *p* < .0001), such that awareness was significantly higher for inferences made about in-group members relative to out-group members.

Overall, these baseline measures replicate the findings previously reported in Payne et al. (2024): participants have a significantly poorer ability to understand out-group minds but show reduced awareness of their accuracy when attempting to understand out-group minds.

#### Effect of Feedback (Part 2)

**Figure 1.**
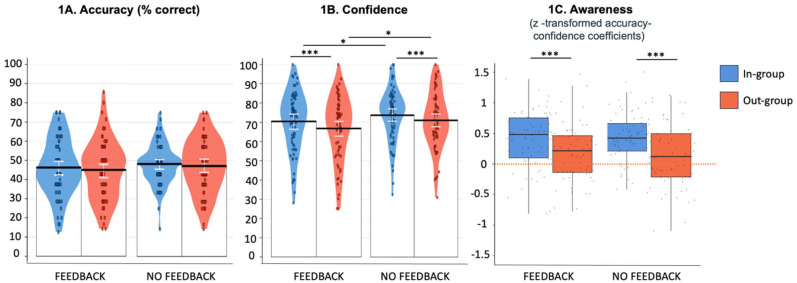
Panels A-C. Performance in Feedback versus No feedback conditions in Experiment 1, for both in-group members and out-group members. (1A) Mean accuracy of mental state inference; (1B) Mean confidence in mental state inferences. In 1A and 1B, coloured segments show smoothed density curves for the full data distribution, while individual dots indicate mean score per participant. (1C) The distribution of *z*-scores representing participants’ awareness of their mental state inference ability. The boxplots illustrate the spread of data, while the points show individual participant data. The dotted orange line represents 0, that is, no correlation between accuracy and confidence. Horizontal bars show post-hoc comparisons with asterisks denoting significance, as determined via likelihood ratio tests (see ‘Results’).

Figure 1 displays participants’ performance on test trials according to whether they received Feedback.

#### Part 2: Accuracy

The generalised linear mixed model testing accuracy rates revealed that neither the main effects (Group Status; *p* = .288, Condition; *p* = .330) nor their interaction (*p* = .910) were significant (see Figure 1A). Results therefore provide no evidence that provision of feedback increased the accuracy of mental state inferences. It should be noted that the lack of an effect of group status within the test trials is likely due to how these trials were selected, with 4 previously accurate and 4 previously inaccurate trials selected for both in- and out-group targets. Given that trials were matched on previous accuracy across in- and out-group targets (i.e. 50% accuracy for both), and that feedback condition did not impact accuracy, we would not expect a difference between in- and out-group trials to emerge.

#### Part 2: Confidence

The linear mixed model testing confidence ratings revealed a significant main effect of Condition (χ²(1) = 4.718, *p* = .029), such that confidence was significantly reduced for participants who had received feedback, compared to those who had not (see Figure 1B). There was also a significant main effect of Group Status (χ²(1) = 14.277, *p* = .0002), such that participants were significantly less confident about out-group trials than in-group trials. The interaction between Condition and Group Status was not significant (*p* = .614). These results suggest that the effect of receiving feedback about previously incorrect inferences was to significantly reduce participants’ confidence in their ability to make accurate inferences on new trials.

#### Part 2: Awareness

Analysis of awareness scores revealed a significant main effect of Group Status (*F*(1, 270) = 23.795, *p* < .001), indicating that participants had significantly poorer awareness of their ability to make accurate inferences on out-group trials, relative to in-group trials (see Figure 1C). Neither the main effect of Condition (*p* = .424), nor the interaction between Condition and Group Status was significant (*p* = .905). These results suggest that receiving feedback did not help to improve participants’ awareness of the accuracy of their inferences.

### Discussion

Experiment 1 demonstrated that providing participants with feedback on a subset of their previously incorrect mental state inferences did not significantly improve the accuracy of future inferences. Feedback did reduce participants’ confidence in further trials, but this reduction in confidence did not translate into improved awareness of the accuracy of their mental state inferences, as assessed by the correlation between participants’ confidence in their inferences and the accuracy of those inferences. These results suggest that feedback that allows a participant to learn about the accuracy of their inferences about an individual mind does not result in more accurate mental state inferences about other group members or an increased awareness of the accuracy of those inferences.

It should be noted, however, that there may be methodological reasons why the effect of feedback failed to generalise to inferences about other group members. First, the feedback was very limited in scope (only 8 of the original 56 trials) and therefore may not have been sufficient for participants to develop a better understanding of others’ minds. Second, participants may have assumed that the remaining 48 trials on which they did not receive feedback were accurate, leading to a lack of motivation to reconsider their inferences. Finally, the feedback provided was exclusively negative and evenly distributed across in-group and out-group trials. As a result, the feedback was not truly representative of their baseline performance, which showed significantly higher accuracy for in-group trials compared to out-group trials.

As a consequence of these methodological factors, Experiment 2 aimed to provide feedback to participants that was representative of their overall performance across all trials. Moreover, we tested whether receiving feedback about specific targets led to improvement in understanding that individual target, as opposed to a general improvement in understanding the target’s group.

## Experiment 2

Experiment 2 largely followed the design of Experiment 1, but participants were provided with feedback on all of their mental state inferences about both in-group and out-group targets. Participants were informed whether their initial inference was correct or incorrect but given no further information. When incorrect inferences had been made, participants were able to update their mental state inference. Such a design allows it to be determined whether provision of feedback about an individual’s mind allows improvement in mental state inferences about that particular mind, and whether any improvement differs according to whether the mind represents an in-group or out-group target.

We hypothesised (see pre-registration linked above) that overall accuracy, confidence and awareness would be higher for in- versus out-group targets. If feedback about an individual mind allows one to improve mental state inferences about that particular mind, we hypothesised that accuracy, confidence, and awareness would be increased after, compared to before, feedback. We hypothesised that these effects may differ as a function of group status, allowing that participants may be less willing to update their representation of out-group members compared to in-group members and/or may still have a poorer (more sparse) representation of the variance in out-group views, such that feedback informing one of an incorrect inference about the target’s mental state may be less useful in identifying a correct inference.

### Methods

#### Participants

In Experiment 2, following our pre-registered sampling strategy, we recruited a final sample size of 90 participants (mean age = 45.45 years, *SD* = 14.88 years, age range = 20–94, 47 female and 43 male). This sample size was determined via WebPower for a repeated measures ANOVA with one group and two measures (in-group vs. out-group). Our goal was to obtain .80 power to detect a small-to-medium main effect of group status (.3) at the standard .05 alpha error probability (based on our experience with Experiment 1, we aimed for the more conventional power level of .80 in this experiment). Participants were recruited and tested using the same method and criteria as Experiment 1.

#### Stimuli

From the 56 pairs of statements used in Experiment 1, 40 pairs were selected based on responses from Experiment 1 as those least likely to elicit responses of ‘neither agree nor disagree’ when participants are asked to give their own opinion. This was to reduce the likelihood of participant-level exclusions based on this criterion.

#### Design

Experiment 2 employed a within-subjects design, such that all participants completed a set of baseline trials, all participants received feedback on those trials, and all participants underwent test trials in a single session. As such, there were two within-subject factors: (1) Timepoint (Before Feedback vs. After Feedback) and (2) Group Status (In-group vs. Out-group).

### Procedure

#### Task 1: Assessing Group Status

The first task determined participants’ group status in relation to each of the targets they would be asked to make mental state inferences about and was completed in the same manner as in Experiment 1 but with 40 starting statements.

#### Task 2: Inferring the Mental States of Others

The second task measured participants’ accuracy in inferring the mental states of others, their confidence on each trial, and how aware participants were of their own ability to make accurate inferences, as in Experiment 1. No feedback was given at this stage.

#### Task 3: Receiving Feedback and Updating Inferences

In the final task, participants underwent 40 further trials such that, on each trial, participants were reminded of one of their previous inferences. For instance, ‘Earlier we told you that Participant 437 [the target-mind] said that they *strongly disagree* that they believe in the superiority of their own gender. Based on this information, we asked you to predict Participant 437’s response to the statement that ‘We should ensure that no one is denied a job due to prejudice’. You said that you thought Participant 437 would strongly agree with this statement and were 98% confident in your answer’. For each inference, participants were told whether it had been correct or incorrect, but not what the true answer was. Rather, they were given the opportunity to update their inference, on the same response scale as before and, moreover, were asked to give a confidence score on their final answer, whether updated or not. Again, participants were told that there was the possibility of a bonus payment if they achieved over 75% accuracy, and the ‘attention check’ trials outlined earlier were also included.

#### Exclusion Criteria

In Experiment 2, the pre-registered criteria stated that: (i) whole datasets would be removed and replaced for participants who failed to pass more than one attention check. This exclusion criterion accounted for 2 exclusions; (ii) whole datasets would be removed for participants who, when they opted to ‘change’ answer, kept their previous answer on >25% trials, as this suggests a lack of engagement. This exclusion criterion accounted for 0 exclusions. However, further to the pre-registered criteria, we also excluded (iii) individual trials on which participants, when they opted to ‘change’ answer, kept their previous answer. This accounted for 3.6% of trials. The pre-registered criteria also stated that (iv) participants who, in >25% of trials choose to ‘change’ an already accurate answer, would be excluded as this would suggest a lack of engagement. This exclusion criterion accounted for 0 exclusions. However, further to the pre-registered criteria, we also applied this exclusion at the trial-level, such that (v) trials on which participants choose to ‘change’ an already accurate answer were removed. This accounted for 0.5% of trials. (vi) Trials on which participants kept an inaccurate answer were also excluded. This was because, given the reward incentive, there was no reason to keep an inaccurate answer other than a lack of interest or inattention to the task. This exclusion criterion accounted for 13% of trials. (vii) Trials on which participants responded ‘I neither agree nor disagree’ were excluded and, relatedly, whole datasets were removed for participants who had more than 50% of trials fall under this exclusion criteria. This exclusion criterion accounted for 0 participant-level exclusions but 7% of trials. Further, (viii) whole datasets were removed for participants who had more than 25% of trials with response times <1,000 ms and >30,000 ms. This exclusion criterion accounted for 1 exclusion. (ix) Finally, whole datasets for participants who, after other exclusion criteria had been applied, had <6 viable in-group or out-group trials, were excluded and their data replaced. This exclusion criterion accounted for 2 exclusions. Overall, the whole datasets for 5 participants were excluded and replaced.

#### Analysis Strategy

Unless otherwise stated, all analyses were pre-registered.

Accuracy: Mixed-effect models were used to assess the effects of Group Status (In-group vs. Out-group), Timepoint (Before vs. After feedback), and their interaction on participants’ accuracy scores. The models included ‘participant’ as a random intercept.

A subsidiary analysis, which was not pre-registered, was conducted to test whether any improvement in accuracy exceeded what could be achieved by simply knowing which trials were correct or incorrect. To this end, a chance-level performance rate was calculated for each participant. This calculation involved determining the proportion of correct and incorrect responses for in-group and out-group trials separately, for each participant. For example, for in-group trials, the proportion of correct responses was multiplied by a weighting of 1 (as participants were certain that these responses were correct). If a participant had 45% correct in-group responses, this would be calculated as 0.45 × 1 = 0.45. The proportion of incorrect responses was then multiplied by 0.25, as there was one correct option out of the four remaining choices. If 55% of in-group trials were incorrect, this would be calculated as 0.55 × 0.25 = 0.1375. These values (0.45 and 0.1375) were then added to obtain the chance-level performance for in-group trials (0.588 or 59%). This process was repeated for out-group trials. To determine if participants performed above their own chance-level, two *t*-tests were conducted: one comparing actual accuracy to chance-level accuracy for in-group trials and another for out-group trials.

Confidence: The second planned analysis used mixed effect models to assess the main effects of Group Status (In-group vs. Out-group) and Timepoint (Before vs. After feedback) and their interaction on participants’ confidence scores. The model included ‘participant’ as a random intercept.

Awareness: The final planned analysis determined whether awareness (correlation between accuracy and confidence) differed as a function of Group Status (In-group vs. Out-group), Timepoint (Before vs. After feedback) or their interaction. The correlation between each participant’s accuracy and confidence scores at each timepoint (Before vs. After feedback), for both in-group and out-group trials, was calculated. All correlation scores were *z*-transformed and entered into a repeated measures ANOVA with factors of Group Status and Timepoint.

### Results and Discussion

**Figure 2.**
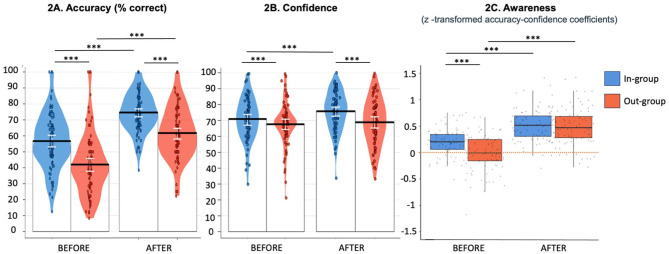
Panels A-C. Performance Before versus After feedback in Experiment 2, for both in-group members and out-group members. (2A) Mean accuracy of mental state inference; (2B) Mean confidence in mental state inferences. In 2A and 2B, coloured segments show smoothed density curves for the full data distribution, while individual dots indicate mean score per participant. (2C) The distribution of *z*-scores representing participants’ awareness of their mental state inference ability. The boxplots illustrate the spread of data, while the points show individual participant data. The dotted orange line represents 0, that is, no correlation between accuracy and confidence. Horizontal bars show post-hoc comparisons with asterisks denoting significance, as determined via likelihood ratio tests (see ‘Results’).

Figure 2 displays participants’ accuracy, confidence and awareness before and after receiving feedback in Experiment 2.

#### Accuracy

The generalised linear mixed model on accuracy data revealed a significant main effect of Timepoint (χ²(1) = 213.39, *p* < .0001), such that accuracy was significantly higher after feedback than before feedback, and a main effect of Group Status (χ²(1) = 118.07, *p* < .0001), such that accuracy was significantly higher for in-group trials relative to out-group trials. The interaction between Timepoint and Group Status was not significant (*p* = .89). These results replicated our previous finding of greater accuracy for in- versus out-group trials (Payne et al., 2024) and suggested that receiving feedback helped to improve inference accuracy, regardless of the target’s group status.

To test whether this improvement in accuracy exceeded what would be expected by chance, two t-tests were conducted, the first comparing participants’ actual performance relative to chance level performance in in-group trials and the second for out-group trials. These analyses revealed that participants were performing significantly above chance in in-group trials (*t*(89) = 24.729, *p* < .0001), as well as in out-group trials (*t*(89) = 26.211, *p* < .0001).

#### Confidence

The linear mixed model testing confidence ratings revealed a significant main effect of Timepoint (*X*^2^(1) = 41.766, *p* < .0001) such that confidence was significantly higher after receiving feedback than before it. There was also a significant main effect of Group Status (*X*^2^(1) = 63.216, *p* < .00010), such that participants were significantly less confident about out-group trials than in-group trials. A significant two-way interaction between Group Status and Timepoint (χ²(1) = 5.555, *p* = .0184) on confidence scores was observed. Post-hoc comparisons with Bonferroni correction revealed that participants were significantly more confident about in-group trials, relative to out-groups, both before (*p* = .0003) and after feedback (*p* < .0001). Confidence increased for in-groups after receiving feedback (*p* < .0001) but did not increase for out-groups (*p* = .181). Given that participants had, on average, higher accuracy for in-group trials, most will have received more positive feedback about in-group trials relative to out-group trials, which may explain why confidence increased for in-group trials only.

#### Awareness

The analysis revealed a significant main effect of Timepoint on awareness (*F*(1, 356) = 135.359, *p* < .0001), with awareness significantly higher after feedback compared to before feedback. There was also a significant main effect of Group Status (*F*(1, 356) = 10.067, *p* = .0016), with higher awareness levels for in-group trials, compared to out-group trials. These main effects were qualified by a significant interaction between Timepoint and Group Status (*F*(1, 356) = 5.982, *p* = .015). Awareness levels were significantly lower for the out-group compared to the in-group before feedback (*p* < .0001), but there was no significant difference between the out-group and in-group after feedback (*p* = .956). Post-hoc comparisons additionally showed that awareness improved significantly after feedback – compared to before feedback – for both in-group (*p* < .0001) and out-group trials (*p* < .0001).

Experiment 2 demonstrated that providing participants with target-specific feedback that gives a representative understanding of their ability to make accurate inferences for in- and out-groups improves their accuracy on future inferences about those target individuals, as well as improving their awareness of their ability to do so. In addition, awareness was improved more for out-group than for in-group targets. This differential effect of feedback on awareness makes it unlikely that the effect of feedback is purely motivational, as might otherwise be proposed, given its equal effect on accuracy.

## General Discussion

This study aimed to examine whether receiving feedback about the accuracy of one’s mental state inferences could improve those inferences and help to recalibrate confidence in one’s ability to make accurate inferences, for both in- and out-groups. In Experiment 1, we tested whether feedback about one’s accuracy of inferences for in- and out-group targets would generalise to other members of the target group but found no effect of this feedback on the accuracy of participants’ inferences, or their awareness of their accuracy. It should be noted, however, that feedback reduced participants’ general level of confidence in their mental state inferences. In contrast, Experiment 2 demonstrated that receiving feedback that is representative of one’s ability (both positive and negative feedback on inferences made about individual targets) allowed participants to significantly improve their mental state inferences for both in- and out-group members. Importantly, Experiment 2 also demonstrated that feedback improved participants’ awareness of their mental state inference accuracy for both in- and out-groups. After receiving feedback, participants revised their original inferences and provided new confidence ratings for these updated answers. While it remains possible that these confidence ratings were also influenced by memory of the positive or negative feedback received on participants’ initial responses, our awareness measure captures how well participants’ new confidence ratings align with the accuracy of their revised inferences. Thus, the observed increase in awareness reflects an improvement in the calibration between inference accuracy and confidence, rather than simply reduced confidence in previously incorrect inferences. If memory of prior feedback does contribute, it would likely serve to enhance rather than undermine this calibration, but we acknowledge that the precise mechanism of improvement warrants further investigation. Critically, this improvement in awareness was greater for out-group members: while participants initially showed poorer awareness (confidence-accuracy alignment) for out-group inferences, feedback eliminated this disparity, bringing out-group awareness up to the same level as in-group awareness.

Experiment 1 utilised a mixed design, with participants assigned to either the feedback or no-feedback group. In contrast, Experiment 2 utilised a within-participants design, comparing scores before and after feedback. This difference between experiments was necessitated by the hypotheses under examination: specifically, as Experiment 1 was looking at whether feedback generalised across targets, it was not possible to use a within-participants design in this experiment. Similarly, we could not analyse Experiment 1 using a within participants factor of before versus after feedback, because the design meant that the trials chosen for Part 2 were equally distributed between those on which participants were previously accurate and inaccurate, for both in- and out-groups. Thus, a before versus after feedback analysis would not have been meaningful, as all participants would have had 50% accuracy for both in- and out-group before feedback for these trials. Although in theory some of the differences in results between the experiments could be due to this difference in design, this is unlikely given that participants were matched on Part 1 accuracy, confidence and awareness across the feedback and no-feedback groups in Experiment 1.

Overall, these results show that people were willing and able to improve their understanding of individual others if they received feedback on the accuracy of their mental state inferences, even for out-group target minds. While previous studies have shown that inference accuracy can be improved with corrective feedback (Marangoni et al., 1995), this is the first study to show that such feedback can also improve people’s understanding of out-group views and, critically, recalibrate people’s awareness of their (in)ability to understand out-groups. This finding is particularly important because the study used real-world targets. In contrast, most previous tasks that assess theory of mind ability use fictional scenarios (Baron-Cohen et al., 1985; Dziobek et al., 2006; see also Long et al., 2022; Marangoni et al., 1995) in which the accuracy of mental state inferences is evaluated against responses deemed ‘correct’ by consensus (e.g. a group of raters) or by logic, rather than against a real-world ground truth. This lack of an objective benchmark is problematic because feedback about one’s performance in tasks that contain fictitious characters – who do not have real mental states – is presumably less likely to alter one’s representation of the minds of others, as there is no objective standard against which to judge accuracy. Such a lack of an objectively correct answer also means that claims of increased accuracy are hard to justify.

Experiment 1 provided no evidence that feedback about the mental states of individual targets improves the accuracy of mental state inferences for other members of the target’s group. Several methodological features may explain this result, and it is possible that an increased amount of feedback, about more individuals, may result in generalisation from individual target members to other group members. It should be noted, however, that the existence of heterogeneity with respect to mental states present in both in- and out-groups means that there exists a limit as to how useful feedback from individuals will be in predicting the views of other group members.

Experiment 1 did show an effect of feedback on the confidence with which participants made mental state inferences. In real-world scenarios, this effect may be extremely beneficial if it results in individuals making fewer assumptions about out-group views. If the effect of the reduced confidence observed after feedback is that individuals ask out-group members their views, rather than incorrectly assuming more extreme views than out-groups really hold (Ahler & Sood, 2018) then out-groups may be better understood and affective polarisation reduced as a consequence of feedback. Indeed, previous studies have shown that people are more likely to gather extra information when their confidence is low (Desender et al., 2018, 2019), and Pennycook et al. (2021) have also demonstrated that when people are motivated to consider the accuracy of information (e.g. social media posts), they subsequently share less false information. Thus, another potential benefit to acquiring feedback about one’s accuracy over time is that people may be less inclined to share their inaccurate assumptions about out-group views with others.

Unfortunately, prior work offers limited evidence for direct, causal effects of improvements in ‘interpersonal accuracy’ on social or behavioural outcomes (Schmid Mast & Hall, 2018). However, we view this largely as a measurement and design issue, not necessarily as evidence that changes in inference accuracy, or awareness of such, will lack consequences. Most prior studies treat interpersonal accuracy as a stable, person-level ability that is constant across targets, and not – as we show here – an ability which is target dependent (e.g. differing for in- vs. out-group members). Therefore, without aligning outcome measures to the group membership of the targets on which feedback was given, links between accuracy and social outcomes are likely to appear unclear. Moreover, the wider literature on ‘interpersonal accuracy’ often conflates mental-state inference with perceptual processes such as emotion recognition from facial expressions, making outcome measures hard to interpret because multiple cognitive processes are involved.

In terms of limitations here, it should be noted that a number of features of the task used in this study lack ecological validity. Perhaps most interestingly, feedback was provided in an impersonal fashion rather than via an in-group or out-group member, as may occur in real life. While current data suggests no differences between social and non-social learning from anonymous individuals (Schuster et al., 2022), it is possible that individuals will be less motivated to engage with, or trust, feedback from an out-group member than from an in-group member. In general, the provision of feedback by another individual may make it more likely that feedback will be engaged with due to social pressure, but it is also not clear how frequently we receive direct feedback about the accuracy of our mental state inferences in real-life interactions. A further feature of the study that sometimes departs from real life is that participants were motivated by financial reward to increase the accuracy of their mental state inferences. While this may be the case in any financial transaction with out-group members, and while making accurate inferences about the mental states of others can be of use when either cooperating or competing with others (Tsoi et al., 2016; Yoshida et al., 2010), evidence suggests that individuals are more likely to withdraw from contact with out-groups (Skitka et al., 2005, 2013), meaning that they may be less motivated to make accurate inferences about out-group mental states due to less frequent contact.

Finally, targets were identified as being members of the out-group by a single difference in opinion. Although, typically, people might perceive a person to be out-group on one or more of many different dimensions that did not feature here (e.g. by speaking in a different language or with a different accent, by physical features which identify them as of another race, gender, or from a different geographic origin), seminal work on social identity theory (Tajfel, 1974) has demonstrated that group identities – and subsequent discrimination – can be formed on the basis of assignment to ‘minimal’ groups, for example via preferences for abstract art (Tajfel et al., 1971). If group identity can be generated based on such minimal manipulations, and since the group assignment in the current study was based on differences in opinion for politically polarising topics, we presume that the assignment in the current study should produce effects of group belonging as strong as, or stronger than, those typically seen for minimal group paradigms. However, whether the current results would hold for individuals judged to be out-group members for these other reasons mentioned above is yet to be determined.

Despite these limitations, this study demonstrates that, with feedback, people can learn to make more accurate mental state inferences for both in- and out-group members. Furthermore, feedback allows individuals to become increasingly aware of the accuracy of their mental state inferences. It remains to be established whether this increased awareness about out-group inferences generalises to further out-group members. However, it is reasonable to expect that the more instances in which a person has accurate awareness of their (in)ability to understand an individual member of an out-group, the more their awareness will generalise to other members of the group over time. This study, therefore, constitutes a critical first step in alleviating the incorrect assumptions people hold about out-group members, which could, over time, help reduce polarisation between groups.

## Supplemental Material

sj-docx-1-qjp-10.1177_17470218251404419 – Supplemental material for Feedback on Mental State Inferences Improves Accuracy and Awareness Supplemental material, sj-docx-1-qjp-10.1177_17470218251404419 for Feedback on Mental State Inferences Improves Accuracy and Awareness by Bryony Payne, Geoffrey Bird and Caroline Catmur in Quarterly Journal of Experimental Psychology
