# Supplementary material for: Feedback on Mental State Inferences Improves Accuracy and Awareness
Source: Q J Exp Psychol (Hove). 2025 Nov 24;79(8):2058–72. doi: 10.1177/17470218251404419 (PMC13400811; doi:10.1177/17470218251404419)
Supplement: sj-docx-1-qjp-10.1177_17470218251404419 – Supplemental material for Feedback on Mental State Inferences Improves Accuracy and Awareness [file sj-docx-1-qjp-10.1177_17470218251404419.docx]

Supplemental Materials for:

**Feedback on mental state inferences improves accuracy and awareness**

Bryony Payne^1^, Geoffrey Bird^2,3^, and Caroline Catmur^1^

^1^Department of Psychology, Institute of Psychiatry, Psychology and Neuroscience, King’s College London, UK

^2^ Department of Experimental Psychology, University of Oxford, UK

^3^ Centre for Research in Autism and Education, Institute of Education, University College London, UK

Corresponding author: Dr Caroline Catmur, Department of Psychology, Institute of Psychiatry, Psychology and Neuroscience, King’s College London, UK. caroline.catmur@kcl.ac.uk

Full list of statements used in Study 1 and (in italics) those reused for Study 2.

This study used a subset of 56 statements selected from the Survey of Beliefs and Opinions (See: Saucier, G. (2018). Survey of Beliefs and Opinions (SBO). https://doi.org/10.7910/1DVN/WV55BYC, Harvard Dataverse, V1, UNF:6:6BSBTPItRnlqZ1H8w8Ypog==[fileUNF]).

Note: Statement ‘a’ in each couplet was used as the ‘starting statement’, while statement ‘b’ was used as the ‘target statement’.

*1a. All forms of government are oppressive and undesirable and should be abolished*

*1b. People have a right to maintain private property*

2a. Ethnic groups should be represented proportionally in the nation's power structures

2b. Women should have equal representation in all decision-making structures of our society

*3a. Everyone should pay the same "flat" tax rate, no matter their income*

*3b. I believe in and support the principles of the political right*

*4a. Foreigners and immigrants have a beneficial effect on our society*

*4b. People of different ethnic backgrounds should not have sexual relations with one another*

5a. God is all-powerful, and those whom God chooses will be saved by God's grace alone

5b. All events are predetermined

*6a. I am devoted to the principles and interests of the church*

*6b. Nothing is ever predetermined by fate or divine providence*

*7a. I am not devoted to any church*

*7b. The human being has a spiritual rather than an animal nature*

8a. I am opposed to constitutional forms of government

8b. Because capitalism benefits the upper classes more than the lower classes, it must eventually be abolished

*9a. I am strict in moral and religious matters*

*9b. There is a struggle in the universe between the forces of good and the forces of evil*

10a. I believe in a messiah

10b. I disapprove of the principles held by those on the political right

*11a. I don't believe in redistributing property so that each individual has an equal share*

*11b. Industrial factories should be owned by the government*

12a. I don't believe that any person receives special enlightenment

12b. There's little use in emphasizing the spiritual aspect of being

*13a. I emphasize reason, scientific inquiry, and human fulfilment in the natural world*

*13b. I don't believe in a messiah who will come to save the world*

*14a. I favour free intellectual inquiry*

*14b. The account of the creation of the universe given at the beginning of the Bible is literally true*

*15a. I favour government ownership of all media (radio, television, newspapers, internet)*

*15b. We must abolish capitalism*

*16a. I favour human rather than religious values*

*16b. Adults should be discouraged from experimenting with unusual forms of sexual behaviour*

*17a. I feel that basically the world is not a fair place*

*17b. The people running this country don't really care what happens to people like me*

18a. I have little interest in going "back to Nature" (living a simple life in a natural setting)

18b. Animals don't have souls or spirits

19a. I prefer, and I am devoted to, the United States and its institutions

19b. Capitalism ought to be overthrown by a revolution of the poor people in the working class

*20a. I question the belief that there is a personal God who created and rules the world*

*20b. Population growth ought to be controlled*

*21a. I see little value in working for peace, human rights, and social justice*

*21b. I favour protecting the environment from destruction and pollution*

*22a. I support the traditional institutions of marriage and family*

*22b. This nation's military budget should be reduced drastically*

*23a. Interracial dating should be avoided*

*23b. I am militant in my devotion to and glorification of my country*

*24a. It is right that same-sex marriages are legal and acceptable*

*24b. Government-supported social welfare programs have a bad effect on society*

*25a. Men should have power over women*

*25b. Deceit and manipulation are justified in pursuing and maintaining power in the political world*

*26a. Not everything can be explained in terms of matter, physical events, and science*

*26b. There is no God or gods*

*27a. One's status and one's political power ought to be based on how much property and wealth one has*

*27b. People of different races and nationalities should live in different places apart from one another*

*28a. Our public institutions have become corrupt*

*28b. Our national government is too dependent on foreigners*

29a. People who earn wealth should always have the right to keep it

29b. Government should function only to punish crimes, enforce contracts, and provide national defence

*30a. Poor people are poor because of bad attitudes*

*30b. We should ensure that no one is denied a job due to prejudice*

*31a. Poor people are poor because of prejudice and discrimination*

*31b. I am opposed to the death penalty (execution) as a punishment for crimes*

*32a. Poor people are poor because of the low wages provided by business and industry*

*32b. The government ought to take responsibility for the individual and social welfare of its citizens*

*33a. Possession and use of drugs should not be considered a crime*

*33b. Sexual freedom is damaging to society*

*34a. Present nations must be eliminated so that nations can join together in a global economic and political union*

*34b. I favour redistributing wealth so as to benefit poor and disadvantaged people*

*35a. Private ownership (of land, capital, and machinery) must be ended*

*35b. I oppose technology*

*36a. Religion should play the most important role in civil affairs*

*36b. A particular set of beliefs is superior to all others*

*37a. Religious considerations should be excluded from civil affairs and public education*

*37b. I reject military virtues and ideals*

*38a. Religious organizations should be taxed in the same way as any business*

*38b. We must ensure that women have access to legal abortions*

*39a. Religious teachings should be a central part of public education*

*39b. The only birth-control method permitted should be abstinence from sexual activity*

40a. Science gives a better account than religious scriptures of how the universe was created

40b. If a so-called crime has no victim (as with drugs or prostitution), it should not be a crime

*41a. Sons should obey their fathers without question*

*41b. I believe the Bible is the only authority and is infallible*

*42a. The government does not have any responsibility for the individual and the social welfare of individual citizens*

*42b. Government agencies should not intervene in the economy*

*43a. The government ought to suppress and censor the opposition*

*43b. Heterosexual people ought to have more privileges than gay or homosexual people*

*44a. The government should redistribute wealth fairly to all of the people*

*44b. The capitalist system will inevitably outlast any socialist system*

45a. The justice system is too soft on criminals

45b. We should reduce the influence of foreigners on our government and our culture

46a. The marriage of a man and a woman is sacred and holy

46b. Prostitution should not be considered a crime

47a. There is only one God

47b. Moral laws are fixed, absolute, and the same everywhere

48a. There ought to be more study of the scriptures and more prayer meetings

48b. A woman must honour and obey her husband and her husband's family

*49a. There should be strong punishment of those who break God's laws about abortion, pornography, and marriage*

*49b. War is often the only means of preserving the honour of the nation*

*50a. This nation's military needs must take priority over other needs*

*50b. Gay or homosexual people should be kept away from children*

*51a. Two people should have freedom to engage in any form of sexual behaviour they both desire*

*51b. We should have severe laws against the sales and use of drugs like marijuana, cocaine, and heroin*

*52a. We need tough leaders who can silence the troublemakers and restore our traditional values*

*52b. Some minority groups have not earned the right to be treated equally*

53a. We ought to take power away from local governments and give more power to our national government

53b. We should make incomes more equal, no matter the job or profession

*54a. We should be suspicious about international organizations (such as the United Nations)*

*54b. Our nation is being damaged by excessive concern for minorities*

55a. We should increase spending that will build up our nation's armed forces

55b. Voters who fail to mark their ballot according to instructions have no right to have their vote counted.

*56a. When I experience doubts about religion, I feel upset*

*56b. I adhere to an organized religion*
